# Cardiac T1 mapping in congenital heart disease: bolus versus infusion protocol for measurement of myocardial extracellular volume

**DOI:** 10.1186/1532-429X-17-S1-W22

**Published:** 2015-02-03

**Authors:** Nadya Al-Wakeel, Sanaz Rastin, Frédéric H Münch, Felix Berger, Titus Kuehne, Daniel Messroghli

**Affiliations:** 1 German Heart Institute Berlin, Berlin, Germany

## Background

Measurement of myocardial extracellular volume fraction (ECV) with T1 mapping cardiac magnetic resonance (CMR) before and after the application of a gadolinium-based extracellular contrast agent enables the assessment of diffuse myocardial fibrosis. The equilibrium between blood and myocardium contrast concentration required for ECV measurements can be achieved with a primed contrast infusion (equilibrium contrast-CMR). In healthy volunteers it could be shown that a single bolus may also be sufficient to reach equilibrium. We hypothesized that equilibrium between blood and myocardium contrast distribution can be achieved with the bolus-only technique to accurately measure diffuse myocardial fibrosis in patients with congenital heart disease (CHD).

## Methods

A study group of 23 patients with CHD (age range 14 - 45 years, mean age 25.8 years) was compared with 17 healthy volunteers (age range 23 - 30 years; mean age 25.1 years). Using modified Look-Locker inversion recovery (MOLLI) T1 mapping before application, 15 minutes after bolus injection, and during constant infusion of Gd-DOTA, T1 values were obtained for blood pool and myocardium of the interventricular septum (IVS), the left ventricular (LV) antero-/lateral wall, and the inferior or anterior wall of the right ventricle (RV) in a single midventricular plane in short axis or in transverse orientation.

## Results

**Figure 1:**
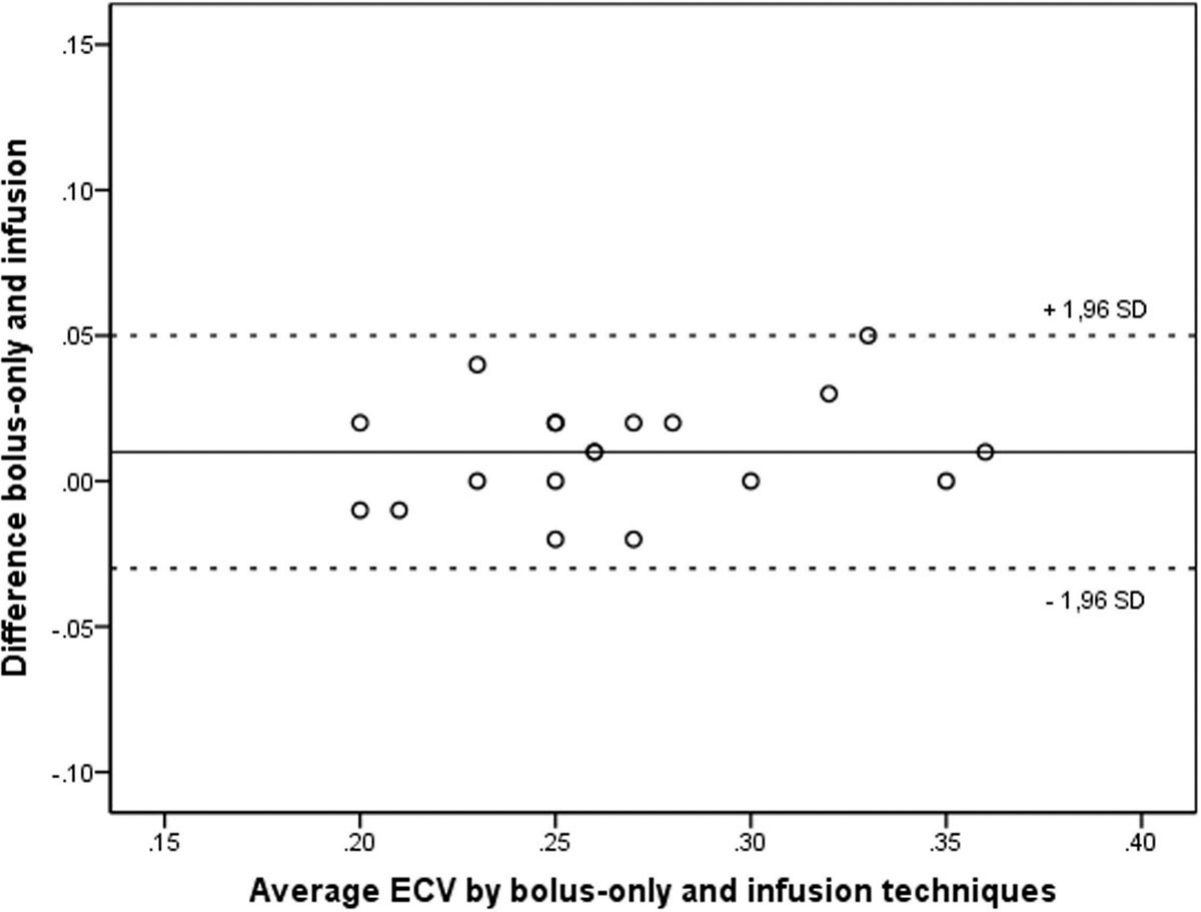
Bland-Altman analysis of ECV by bolus-only and infusion techniques in patients with CHD (LV in transverse orientation)

In CHD patients, correlation between ECV by bolus-only and infusion was strong for IVS, LV and RV (R2 = 0.88, 0.85, and 0.79, respectively). ECV of IVS, LV and RV by both techniques correlated strongly in healthy volunteers (R2 = 0.91, 0.81, and 0.74. respectively). Bland-Altman plots did not show significant bias between the techniques in patients and healthy volunteers for any of the analyzed regions (Figure 1).

## Conclusions

Based on T1 mapping, ECV of LV and RV myocardium can be measured accurately by the bolus-only technique in patients with CHD. The use of a bolus-only approach facilitates the integration of ECV measurements into clinical CMR routine workflow across a wide range of CHD.

## Funding

The study was funded by the German Research Foundation (DFG ME 3508/4).

